# Pembrolizumab-Associated Hypoparathyroidism: A Single Case Report

**DOI:** 10.1016/j.aace.2020.11.003

**Published:** 2020-11-28

**Authors:** Israa Mahmood, Nitesh D. Kuhadiya, Michael Gonzalaes

**Affiliations:** 1 Division of Endocrinology, Diabetes and Metabolism, Renown Health, Reno, Nevada; 2 Renown Health & Diabetes & Endocrine Center of Nevada (DECON), Reno, Nevada

**Keywords:** pembrolizumab, hypoparathyroidism, hypocalcemia, PTH, parathyroid hormone

## Abstract

**Objective:**

To evaluate a case of pembrolizumab-induced hypoparathyroidism leading to hypocalcemia.

**Methods:**

The diagnostic tests performed included calcium and parathyroid hormone level detection and calcium-sensing receptor gene analysis.

**Results:**

A 71-year-old Caucasian man was diagnosed with stage IIIB adenocarcinoma of the lung and received radiation therapy but had no other exposure to radiation. Pembrolizumab 200 mg intravenous every 3 weeks was started 5 years after the initial diagnosis. The patient’s corrected calcium level was 9.2 mg/dL (normal, 8.5-10.5 mg/dL) at the start of pembrolizumab therapy. The calcium level after the 13th dose of pembrolizumab was 8.1 mg/dL (normal, 8.5-10.2 mg/dL), leading to endocrinology referral. The patient’s parathyroid hormone and corrected calcium levels after the 22nd dose were 4.3 mg/dL (normal, 14-72 pg/mL) and 6.5 mg/dL (normal, 8.5-10.2 mg/dL), respectively. He denied symptoms of latent tetany on presentation while on pembrolizumab for 15 months but complained of fatigue and weakness. The patient had no history of autoimmune diseases or neck injuries. Calcium-sensing receptor gene analysis was negative for genetic mutations. Immunotherapy-mediated hypoparathyroidism was diagnosed. He was treated with daily oral calcium carbonate (2000 mg), calcitriol 0.5 μg, 1 dose of calcium gluconate 2 g intravenous, and 3 doses of calcium chloride 1 g intravenous. His fatigue, weakness, and calcium levels improved with therapy.

**Conclusion:**

Pembrolizumab treatment may have resulted in immune-mediated hypoparathyroidism, leading to hypocalcemia. It is important to report such cases to understand its presentation and timing in relation to pembrolizumab, which further facilitates its timely treatment.

## Introduction

Most cases of acquired hypoparathyroidism occur after thyroidectomy or are autoimmune mediated. In adults, most cases occur postsurgically. In a retrospective analysis of adults evaluated in a metabolic bone unit of an endocrinology service in Brazil, over 80% of cases of acquired hypoparathyroidism were postsurgical whereas <20% were autoimmune.^1^ Drug-induced hypoparathyroidism is extremely rare. Here we report a case of hypoparathyroidism and hypocalcemia with pembrolizumab, an immune checkpoint inhibitor used for the treatment of advanced lung cancer.

## Case Report

**Fig. 1:**
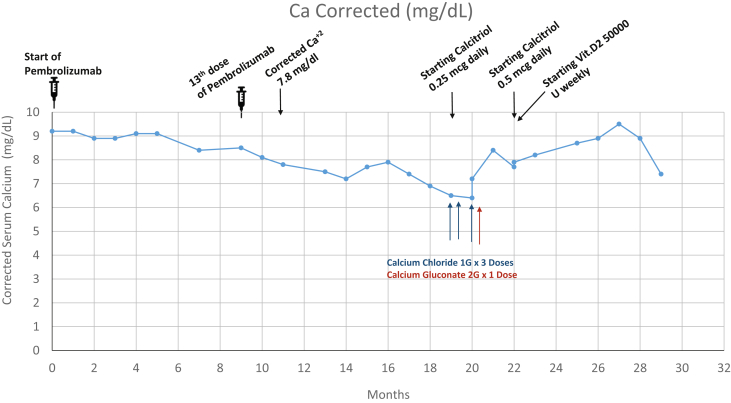
Trend of corrected calcium levels during pembrolizumab therapy and interventions for correction of low calcium levels.

A 71-year-old Caucasian man was diagnosed with a malignant neoplasm of an unspecified part of the right bronchus or right lung (stage IIIB) and a malignant neoplasm of the upper lobe of the left lung and left bronchus. Right lower and upper lobe wedge resection performed soon after diagnosis demonstrated an adenocarcinoma. Two different courses of radiation treatment were performed, one in the left lung and a subsequent treatment in the right lobe of the patient’s lung. Pembrolizumab, 200 mg intravenous every 3 weeks, was started 5 years after the initial diagnosis of cancer after the patient demonstrated a high (100%) expression of programmed death-ligand 1 in an adenosquamous pathology core sample. The patient’s corrected calcium level was 9.2 mg/dL (normal, 8.5-10.2 mg/dL) at the start of pembrolizumab therapy as shown in Figure 1 and the Table. He had no past medical history or other risk factors for hypocalcemia. The calcium level after the 13th dose of pembrolizumab was 8.1 mg/dL (normal, 8.5-10.2 mg/dL), leading to endocrinology referral.

**Table:** Trend of Parathyroid Hormone, Corrected Calcium Levels, and 25-OH Vitamin D Levels in Relation to Pemobrolizumab Therapy

Date	Parathyroid Hormone (14-72 pg/ml)	Corrected calcium (8.5-10.5 mg/dL)	Pemobrolizumab dose No.	25-OH vitamin D levels (30-100 ng/ml)
Jun 2017	…	9.2	1	…
March 2018	…	8.5	13	…
April 2018	…	8.1	14	…
May 2018	…	7.8	15 and 16	…
July 2018	…	7.5	18 and 19	…
August 2018	…	7.2	20	…
September 2018	20.4	7.7	21	…
October 2018	…	7.9	22	35
December 2018	…	6.9	25	…
January 2019	4.3	6.5	26	…
February 2019	7.2	6.4	27	…
February 2019	…	7.2	28	21
March 2019	…	8.4	29	…
April 2019	…	7.7	30	…
April 2019	…	7.9	31	…
May 2019	…	8.2	32	…
July 2019	2.3	8.7	33	…
November 2019	1.2	7.4	34	96

**Fig. 2:**
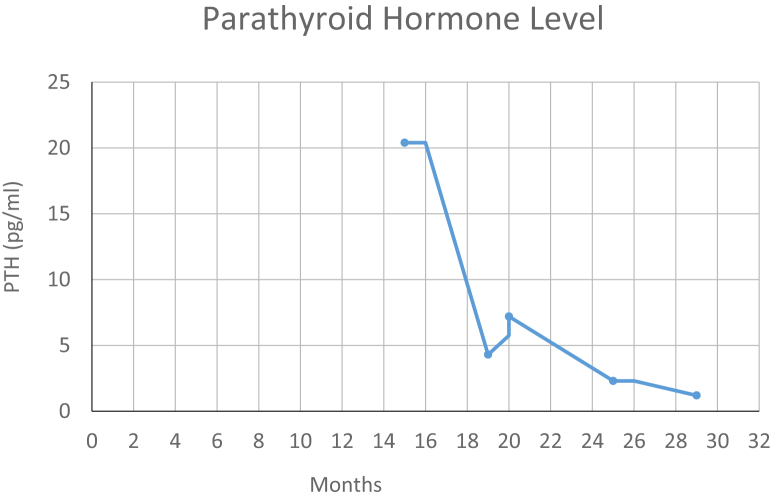
Trend of parathyroid hormone level during pembrolizumab therapy.

The patient’s parathyroid hormone (PTH) and corrected calcium levels after the 18th dose of pembrolizumab were 20.4 pg/mL (normal, 14-72 pg/mL) and 7.7 mg/dL (normal, 8.5-10.2 mg/dL), respectively (Table). After the 22nd dose, the levels were 4.3 mg/dL (normal, 14-72 pg/mL) and 6.5 (normal, 8.5-10.2 mg/dL), respectively (Table). At the time of presentation, the patient had been on pembrolizumab for 15 months. He denied symptoms of latent tetany but complained of fatigue and weakness. His PTH and calcium levels remained low as shown in Figure 2. The patient denied a history of autoimmune diseases, neck injuries, or exposure to neck radiation. Calcium-sensing receptor gene analysis (LabCorp Specialty Testing Group) was negative for any kind of genetic mutation. Pembrolizumab-associated hypoparathyroidism was diagnosed. The patient was started on daily oral calcium carbonate (2000 mg), calcitriol 0.5 μg, 1 dose of calcium gluconate 2 g intravenous, and 3 doses of calcium chloride 1 g intravenous. He continued to receive vitamin D 50 000 IU weekly for 6 months, followed by 50 000 IU every 2 weeks. His fatigue, weakness, and calcium levels improved with therapy (Fig. 2).

## Discussion

To the best of our knowledge, this is the third reported case of pembrolizumab-induced hypoparathyroidism and hypocalcemia. In our case, both PTH and calcium levels started to decrease after the 13th dose of pembrolizumab. In the 2 previously reported cases, the decrease in serum calcium levels was reported after the 15th and the second doses of pembrolizumab, respectively.^2^^,^^3^ Similar events have been recently reported after 1.5 months of concurrent treatment with iplimumab and nivolumab, which are checkpoint inhibitors used for treating melanoma.^4^ Hypocalcemia and hypoparathyroidism were noted in our patient 1.5 months after starting the therapy. It is therefore clear that the dose-response relationship and the timing of pembrolizumab treatment and hypoparathyroidism can vary on a case-to-case basis, emphasizing the need for frequent monitoring of calcium and parathyroid levels.

Fatigue secondary to hypocalcemia was commonly reported in all previously published cases. Fatigue and weakness, if reported, resolved as the calcium level started to normalize in response to treatment. In our patient, calcium levels were stable on oral calcium supplementation, calcitriol, and 50 000 IU per week of vitamin D supplementation for 6 months, followed by 50 000 IU of vitamin D every 2 weeks. A higher requirement of calcium and calcitriol with failure to normalize calcium or the occurrence of unwanted side effects affected tolerability and long-term adherence. Treatment with recombinant human PTH can be considered. One of the limitations of our case report is that we did not have ionized calcium levels available prior to the start of pembrolizumab treatment; however, with the available data on corrected calcium and PTH levels, the clinical message is very clear.

Pembrolizumab is effective for the treatment of various advanced malignancies such as lung cancer as monotherapy and in combination with other chemotherapy agents.^5^ However, it has been shown to induce immune-related side effects, including disturbances of the endocrine system, and most commonly leads to adrenal insufficiency, pituitary hypophysitis, and thyroid dysfunction.^5^ Hypoparathyroidism from immune checkpoint inhibitors is rare. So far, a total of 3 cases, including our case, describing hypoparathyroidism associated with pembrolizumab have been reported. Proposed mechanisms include autoimmune hypoparathyroidism caused by increased T-cell activity against parathyroid tissue or the development of calcium-sensing receptor autoantibodies that inhibit PTH secretion. Vigilance is warranted to facilitate timely recognition and the treatment of immune checkpoint inhibitor-related hypoparathyroidism. It is not clear whether the hypoparathyroidism will be permanent after the discontinuation of pembrolizumab treatment or will resolve over time. Further research and/or case studies are needed to advance our understanding of this relationship and its mechanisms.

## Conclusion

Pembrolizumab may result in immune-mediated hypoparathyroidism leading to hypocalcemia. We recommend timely recognition and treatment of this endocrine disturbance.

## Author Contributions

I.M. and N.D.K. contributed equally to this work.

## Disclosure

The authors have no multiplicity of interest to disclose.
